# Roles of Brain Angiotensin II in Cognitive Function and Dementia

**DOI:** 10.1155/2012/169649

**Published:** 2012-12-11

**Authors:** Masaki Mogi, Jun Iwanami, Masatsugu Horiuchi

**Affiliations:** Department of Molecular Cardiovascular Biology and Pharmacology, Ehime University Graduate School of Medicine, Shitsukawa, Ehime, Tohon 791-0295, Japan

## Abstract

The brain renin-angiotensin system (RAS) has been highlighted as having a pathological role in stroke, dementia, and neurodegenerative disease. Particularly, in dementia, epidemiological studies indicate a preventive effect of RAS blockade on cognitive impairment in Alzheimer disease (AD). Moreover, basic experiments suggest a role of brain angiotensin II in neural injury, neuroinflammation, and cognitive function and that RAS blockade attenuates cognitive impairment in rodent dementia models of AD. Therefore, RAS regulation is expected to have therapeutic potential for AD. Here, we discuss the role of angiotensin II in cognitive impairment and AD. Angiotensin II binds to the type 2 receptor (AT_2_) and works mainly by binding with the type 1 receptor (AT_1_). AT_2_ receptor signaling plays a role in protection against multiple-organ damage. A direct AT_2_ receptor agonist is now available and is expected to reduce inflammation and oxidative stress and enhance cell differentiation. We and other groups reported that AT_2_ receptor activation enhances neuronal differentiation and neurite outgrowth in the brain. Here, we also review the effect of the AT_2_ receptor on cognitive function. RAS modulation may be a new therapeutic option for dementia including AD in the future.

## 1. Introduction

The renin-angiotensin system (RAS) in the brain is well known to be involved in systemic blood pressure control, including the regulation of cerebral blood flow [1]. Angiotensin II, a major player in RAS mainly via the angiotensin type 1 (AT_1_) receptor, plays an important role in the pathophysiology of tissue dysfunction [2, 3]; therefore, RAS blockade by AT_1_ receptor blockers (ARBs) and angiotensin converting enzyme inhibitors (ACEIs), which are widely used as antihypertensive drugs, is expected to prevent multiple-organ damage. Cognitive impairment and dementia are common serious health problems that impair quality of life in the elderly. Previous reports indicate the possibility that treatment with antihypertensive agents prevents the impairment of quality of life including cognitive performance [4, 5]. Possible beneficial effects of RAS blockade on cognitive function are also being highlighted in the clinical field [6, 7]. An epidemiological study by Li et al. recently showed that male subjects treated with ARBs exhibited a significant reduction in the incidence and progression of Alzheimer disease (AD) and dementia compared with those treated with ACEIs and other cardiovascular drugs [8]. Moreover, Davies et al. also reported that patients diagnosed with dementia had fewer prescriptions for ARBs and ACEIs. Interestingly, the inverse associations with AD were stronger for ARBs compared with ACEIs [9]. In contrast, Ohrui et al. demonstrated that long-term use of ACEIs may have a protective role against the development of AD, probably through their direct effects on RAS in the brain [10]. In a subanalysis of the Study on Cognition and Prognosis in the Elderly (SCOPE) trial, hypertensives treated with an ARB, candesartan, showed less decline of specific areas of cognitive function such as attention and episodic memory [11]. However, almost all large clinical intervention trials have shown no significant difference in the incidence of dementia between treatment with ARBs or ACEIs and the placebo group. The Ongoing telmisartan alone and in combination with ramipril global endpoint trial (ONTARGET) and the parallel telmisartan randomized assessment study in ACE intolerant subjects with cardiovascular disease (TRANSCEND) trial showed no clear effects on cognitive outcomes [12]. The reason why RAS blockade failed to prevent dementia may be the short-term observation for the long-term preclinical disease stage of dementia; however, the detailed explanation is not clear. Another reason is the selection of hypertensive patients, who have high cardiovascular disease morbidity, in these trials. A large number of these patients are likely to go on to develop dementia, most likely with strong vascular underpinning. In these trials, vascular dementia and AD are not well distinguished because most studies focused on dementia as subanalysis. As described in the review by Kehoe and Passmore, RAS has multifunctional involvement not only in vascular dementia but also in AD [13]. Therefore, in such specific groups with cardiovascular risk, the distinction of dementia subtype is very important in comparing the incidence of dementia.

The effect of angiotensin II on cognition has been examined in basic studies. Although the blood-brain barrier is impermeable for all RAS components, the local brain RAS has possible physiological and pharmacological functions in the neuronal system [14]. Gard reviewed the contradictory role of angiotensin II in memory and learning in animal studies [15]. Angiotensin II enhances memory and learning in rodents [16, 17], but other studies suggest that angiotensin II decreases cognition [18]. To assess the paradoxical effect of angiotensin II on cognitive function, we therefore performed cognitive tests in mice with continuous activation of angiotensin II, using transgenic mice carrying both the human renin and angiotensinogen genes (hRN/hANG-Tg) [19]. Interestingly, the avoidance rate in hRN/hANG-Tg mice did not increase from 14 weeks of age; however, that from 8 to 13 weeks of age tended to be higher than that in wild-type mice. These findings suggest that the acute or subacute effect of angiotensin II may enhance cognitive function, but chronic treatment with angiotensin II may exhaust neural function and result in cognitive impairment. Angiotensin II induces cerebrovascular remodeling, promotes vascular inflammation and oxidative stress, and results in impairment of regulation of cerebral blood flow (CBF) [20, 21]. Moreover, endothelial function in cerebral vessels was impaired in a genetic model of angiotensin-II-dependent hypertension [22, 23]. On the other hand, Lanz et al. showed that angiotensin II induced sustained central nervous system (CNS) inflammation via transforming growth factor- (TGF-)*β* in an experimental autoimmune encephalomyelitis (EAE) mouse model [24]. Furthermore, angiotensin II induced astrocyte senescence, which is involved in age-associated neurodegenerative disease via superoxide production [25]. In contrast, a centrally active ACE inhibitor, perindopril, was reported to prevent cognitive impairment in chronic central hypoperfusion rats [26] and Alzheimer disease model mice [27]. These reports indicate that continuous angiotensin II stimulation impairs cognitive function via stimulation of the AT_1_ receptor with “environmental degradation of neurons” such as a decrease in CBF and an increase in oxidative stress, CNS inflammation, and cellular senescence in the brain. Such multiple stimuli by angiotensin II induce cognitive impairment following neuronal degeneration.

## 2. Effects of Angiotensin II on Amyloid *β* Metabolism and Cholinergic System

There are two major proposed pathomechanisms of AD; the amyloid cascade hypothesis and the cholinergic hypothesis. Amyloid *β* (A*β*) is a 39–42 amino acid peptide, produced by cleavage of amyloid precursor protein (APP) [28]. A*β* (1–42) causes the neurodegenerative abnormalities that lead to clinical AD [29]. Although the effect of angiotensin converting enzyme on A*β* metabolism is one of the hot topics in the relation between RAS and AD [30], it seems that angiotensin II does not directly affect A*β* secretion or secretase activity via activation of the AT_1_ receptor [31]. On the other hand, blockade of RAS may affect A*β* metabolism. For example, an ARB, valsartan, was able to attenuate oligomerization of amyloid *β* peptides into high molecular weight oligomeric peptides [32]. Moreover, treatment with valsartan also disrupted the development of amyloid *β*-mediated cognitive impairment in Tg2576 mice, a model of Alzheimer disease; however, it is reported that this beneficial effect is not observed with treatment with other ARBs. We previously reported that A*β* (1–40) concentration in the brain of ddY mice that underwent intracerebroventricular injection of A*β* (1–40) was significantly decreased by treatment with an ARB, telmisartan [33]. Moreover, Danielyan et al. reported that intranasal administration of losartan exerts direct neuroprotective effects via its A*β*-reducing and anti-inflammatory effects in the central nervous system [34]. These results indicate that treatment with ARBs may have a beneficial effect on A*β*-induced brain injury through unknown mechanisms on A*β* metabolism by angiotensin II inhibition. On the other hand, brain-penetrating ACEIs such as perindopril prevent cognitive impairment in mice with intracerebroventricular A*β* (1–40) injection via attenuation of oxidative stress and hippocampal astrocyte activation [35]. ACE activity is increased in the hippocampus of these AD mice and suppressed by perindopril treatment. Although there is concern that ACEIs may enhance brain A*β* (1–42) deposition from basic research [36] because ACE converts A*β* (1–42), which plays a causative role in the development of Alzheimer disease, to A*β* (1–40) [37], recent pilot clinical trials showed that ramipril inhibits cerebrospinal fluid (CSF) ACE activity, but did not influence CSF A*β* (1–42) and cognition [38]. The effects of other RAS components involving angiotensin-II-generating enzymes on cognition have also been discussed. A*β* clearance is induced by many kinds of degrading enzyme such as neprilysin (NEP), insulin-degrading enzyme, and endothelin-converting enzyme. Angiotensin II is also generated by degradation of angiotensinogen and angiotensin I by tonins, cathepsins, and chymases as well as ACE. Gene polymorphism in cathepsin G, one of the angiotensin generating enzymes, showed no significant association with AD [39]. In our knowledge, no report has examined the relation between tonin, chymase, and dementia; however, inhibition of angiotensin generating enzymes may also inhibit A*β* degradation. Therefore, it is difficult to assess the effect on A*β* metabolism of drugs that inhibit angiotensin II based on degrading angiotensinogen. Further investigation is necessary to understand the relation among angiotensin II, ACE, other degrading enzymes, and A*β* metabolism.

**Figure 1:**
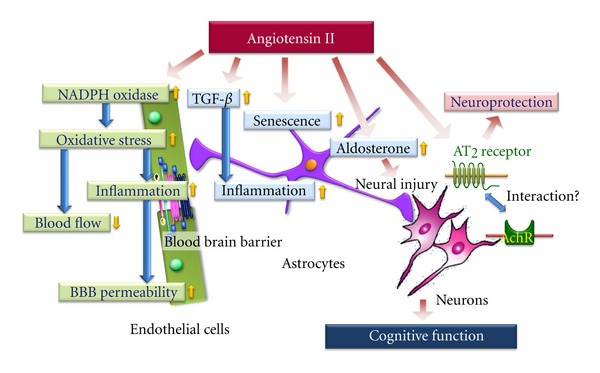
Possible effect of angiotensin II on neurovascular unit. AT_2_: angiotensin II type 2 receptor, AchR: acetylcholine receptor, BBB: blood brain barrier, and TGF-*β*: transforming growth factor *β*.

In the cholinergic hypothesis, AD is also characterized by a loss of neurons, especially those expressing nicotinic acetylcholine receptors (nAChR) [40, 41]. To improve the cognitive deficit in AD, one promising drug target currently under investigation is the neuronal nicotinic alpha7 acetylcholine receptor (*α*7nAChR) [42, 43]. Although there are few reports about the correlation between *α*7nAChR and angiotensin II, Marrero's group has demonstrated that angiotensin II blocks nicotine-mediated neuroprotection against A*β* (1–42) via activation of the tyrosine phosphatase, SHP-1 [44]. They also showed that angiotensin II inhibits *α*7nAChR-induced activation of the JAK2-PI-3 K cascade in PC12 cells through AT_2_ receptor-induced SHP-1 activation [45]. However, AT_2_ receptor-induced SHP-1 activation also induces cerebellar development and neural differentiation [46, 47]. Moreover, A*β* triggered AT_2_ receptor oligomerization in the hippocampus [48] and impaired coupling of the muscarinic acetylcholine receptor (mAChR) to heterotrimeric GTP-binding proteins (G*α* q/11) [49]. Therefore, the AT_2_ receptor may interact with the cholinergic system; however, the actual effect of angiotensin II mediated by AChRs is still an enigma (Figure 1).

## 3. Effects of Angiotensin II on Neurovascular ****Unit

Nonneuronal cells such as vascular cells and glia (astrocytes, microglia and oligodendroglia) comprise the “neurovascular unit” and could play important roles in disease pathogenesis [50]. Especially, CBF functions in concert as a part of the neurovascular unit to maintain homeostasis of the cerebral microenvironment [51]. Iadecola and colleagues demonstrated that angiotensin II increases the production of reactive oxygen species (ROS) in cerebral microvessels via gp91phox (nox-2), a subunit of NADPH oxidase [20, 51]. Moreover, recently they also demonstrated that slow infusion of the pressor angiotensin II induces attenuation of the increase in CBF induced by neural activity (whisker stimulation) and by endothelium-dependent vasodilators, without elevation of mean arterial pressure (MAP) [52]. Such an effect of angiotensin II reduces blood supply and contributes to increased susceptibility to dementia. Interestingly, this angiotensin-induced cerebrovascular dysregulation was attenuated in female compared with male mice [53]. This sexual dimorphism of the cerebral blood-vessel response to angiotensin II may be implicated in the sex difference in cognitive impairment reported in epidemiological studies [54]. On the other hand, Takeda et al. demonstrated that the ARB olmesartan ameliorates amyloid *β*-induced impairment of functional hyperemia evoked by whisker stimulation via a decrease in oxidative stress in brain microvessels [55]. Recently, Zhang et al. reported that angiotensin II increases cerebral microvasculature inflammation via induction of oxidative stress and leads to immune-endothelial interaction, resulting in enhancement of BBB permeability [56]. Therefore, angiotensin-II-induced oxidative stress may have a key role in dysfunction of the neurovascular unit (Figure 1).

On the other hand, several reports indicate the effect of angiotensin II on astrocytes to be neuroinflammation, neuronal damage and astrocyte senescence. For example, Lanz et al. clearly demonstrated that angiotensin II acts as a paracrine mediator, sustaining inflammation in the CNS via TGF-*β* upregulation in astrocytes [24]. We also reported that aldosterone secretion induced by angiotensin II in astrocytes enhances neuronal damage due to angiotensin II [57]. Moreover, Liu et al. showed that angiotensin II induces astrocyte senescence via superoxide production [25]. These findings of astrocyte dysfunction induced by angiotensin II also explain the crucial role of angiotensin II in dysfunction of the neurovascular unit (Figure 1).

## 4. Effect of AT_**2**_ Receptors on Cognition and ****Dementia

**Figure 2:**
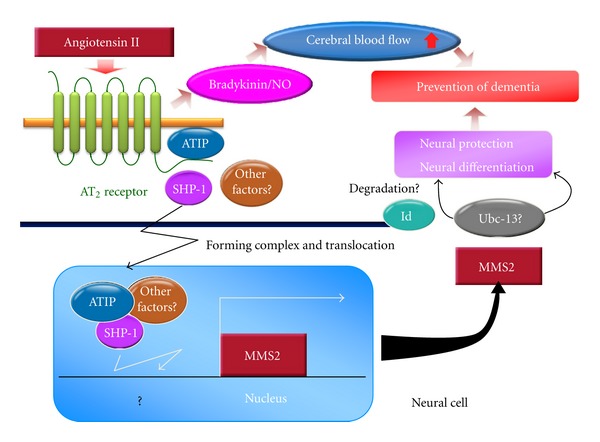
Effect of angiotensin II type 2 receptor signaling on cognitive function. AT_2_: angiotensin II type 2 receptor, ATIP: AT_2_ receptor-interacting protein, Id1: inhibitor of DNA binding protein 1, MMS2: methyl methanesulfonate-sensitive 2, NO: nitric oxide, SHP-1: Src homology 2 domain-containing protein-tyrosine phosphatase 1, and Ubc-13: ubiquitin conjugating enzyme 13.

The major actions of angiotensin II are mediated by the AT_1_ receptor, whereas the role of a second receptor subtype known as the angiotensin II type 2 (AT_2_) receptor is suggested to be protecting of the brain [58]. In the brain, AT_2_ receptors are expressed not only in the vascular wall but also in areas related to learning and control of motor activity [59, 60]. Mice with deletion of the AT_2_ receptor were reported to exhibit worse cognitive function compared with wild-type mice [60]. Reinecke et al. demonstrated the possibility that stimulation of the AT_2_ receptor may promote cell differentiation and regeneration in neuronal tissue [61] and that AT_2_ receptor stimulation supported neuronal survival and neurite outgrowth in response to ischemia-induced neuronal injury [62]. We also demonstrated that AT_2_ receptor signaling enhanced neural differentiation and the repair of damaged DNA through induction of a neural differentiating factor, methyl methanesulfonate-sensitive 2 (MMS2), which is one of the ubiquitin conjugating enzyme variants [47]. Moreover, Gallo-Payet et al. reported that angiotensin II induces neural differentiation and neurite outgrowth via mitogen-activated protein kinase [63] or nitric oxide [64] through AT_2_ receptor activation, and is involved in cerebellar development [65]. Therefore, direct AT_2_ receptor stimulation is expected to have a beneficial effect on cognitive function. We examined the possibility that direct stimulation of the AT_2_ receptor by a newly generated direct AT_2_ receptor agonist, Compound 21 (C21), would enhance cognitive function [66]. Daily intraperitoneal injection of C21 for 2 weeks significantly enhanced spatial learning evaluated by the Morris water maze test in C57BL6 mice, but this effect was not observed in AT_2_ receptor-deficient mice. C21 treatment increased cerebral blood flow assessed by laser speckle flowmetry and hippocampal field-excitatory postsynaptic potential. Moreover, treatment with C21 prevented cognitive decline in an Alzheimer disease mouse model with intracerebroventricular injection of amyloid *β* (1–40). AT_2_ receptor activation is reported to stimulate the release of NO/cGMP and may mediate vascular relaxation and blood flow indirectly through modulation of bradykinin release [67]. In our model, C21-induced cognitive enhancement was attenuated by coadministration of icatibant, a bradykinin B_2_ receptor antagonist. Therefore, direct activation of the AT_2_ receptor improves spatial learning via an increase in microcirculation, partly through modulation of bradykinin. The preventive effect of AT_2_ receptor signaling on dementia is summarized in Figure 2. Clinical use of C21 is expected to be a new therapeutic option in patients with dementia.

## 5. Conclusion

**Figure 3:**
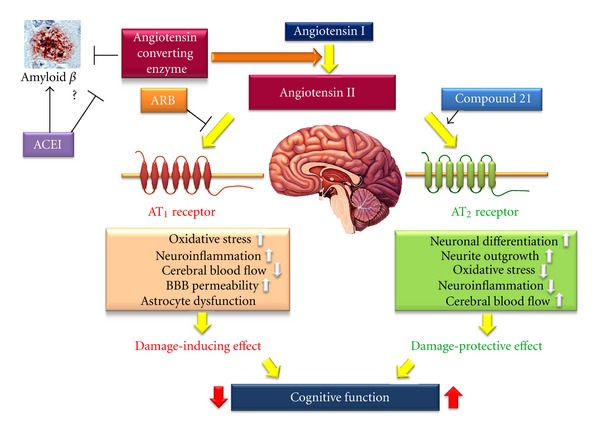
Effect of angiotensin II on cognitive function. ACE: angiotensin converting enzyme inhibitor, AT_1_: angiotensin II type 1 receptor, AT_2_: angiotensin II type 2 receptor, and ARB: angiotensin II type 1 receptor blocker.

Continuous stimulation with angiotensin II may damage neurons via multiple cascades through AT_1_ receptor stimulation. On the other hand, stimulation of the AT_2_ receptor is expected to prevent neural damage and cognitive impairment (Figure 3). However, it is difficult to perform clinical intervention studies to confirm the results of animal studies because of the long-term progression of cognitive impairment. Moreover, in clinical practice, it is not possible to exclude the antihypertensive effect of RAS blockade on cognition in patients with hypertension. However, RAS modulation may be a new therapeutic option for dementia including AD in the future. Therefore, the hypothesis that RAS regulation affects future cognitive function should be confirmed with carefully designed clinical studies.

## Abbreviations

AD: Alzheimer disease
ARB: Angiotensin II type 1 (AT_1_) receptor blocker
AT_1_ receptor: Angiotensin II type 1 receptor
AT_2_ receptor: Angiotensin II type 2 receptor
ACE: Angiotensin converting enzyme
CBF: Cerebral surface blood flow
hRN/hANG-Tg: Human renin and angiotensinogen genes
RAS: Renin-angiotensin system
si: Small interfering
MMS2: Methyl methanesulfonate-sensitive 2.
